# Butyrate induces apoptosis by activating PDC and inhibiting complex I through SIRT3 inactivation

**DOI:** 10.1038/sigtrans.2016.35

**Published:** 2017-02-10

**Authors:** Sha Xu, Cai-Xia Liu, Wei Xu, Lei Huang, Jian-Yuan Zhao, Shi-Min Zhao

**Affiliations:** 1 The Obstetrics & Gynecology Hospital of Fudan University, School of Life Sciences, Shanghai, P.R. China; 2 Institute of Biomedical Science, Fudan University, Shanghai, P.R. China; 3 Collaborative Innovation Center for Biotherapy, West China Hospital, Sichuan University, Chengdu, P.R. China

## Abstract

The underlying anticancer effects of butyrate, an end-product of the intestinal microbial fermentation of dietary fiber, remain elusive. Here, we report that butyrate promotes cancer cell apoptosis by acting as a SIRT3 inhibitor. Butyrate inhibits SIRT3 both in cultured cells and *in vitro*. Butyrate-induced PDHA1 hyperacetylation relieves the inhibitory phosphorylation of PDHA1 at serine 293, thereby activating an influx of glycolytic intermediates into the tricarboxylic acid (TCA) cycle and reversing the Warburg effect. Meanwhile, butyrate-induced hyperacetylation inactivates complex I of the electron transfer chain and prevents the utilization of TCA cycle intermediates. These metabolic stresses promote apoptosis in hyperglycolytic cancer cells, such as HCT116*p53*^*−/−*^ cells. SIRT3 deacetylates both PDHA1 and complex I. Genetic ablation of *Sirt3* in mouse hepatocytes abrogated the ability of butyrate to induce apoptosis. Our results identify a butyrate-mediated anti-tumor mechanism and indicate that the combined activation of PDC and inhibition of complex I is a novel tumor treatment strategy.

## Introduction

Intestinal microbes ferment dietary fibers and produce butyrate, a nontoxic primary energy source for colon epithelial cells. Remarkably, the production of butyrate in intestinal tissue is associated with decreased cancer onset. As a result, butyrate is one of the most intensively studied metabolites, but the underlying molecular mechanisms of butyrate-mediated anticancer properties remain incompletely elucidated. It has been proposed that the ability of butyrate to prevent or treat cancer is potentiated through the induction of apoptosis in various cancer cells. For example, in human gastric cancer cells, butyrate treatment induces death-associated protein kinase (DAPK)-mediated apoptosis.^1^ In addition, in HCT116 colorectal cancer cells, butyrate treatment induces stress response-mediated apoptosis.^2^ In human colon adenocarcinoma cells, butyrate treatment induces apoptosis through up-regulation of B-cell lymphoma 2 (Bcl-2) and inactivation of Bcl-2-associated X protein (BAX).^3^ These mechanisms are plausible because butyrate is a confirmed inhibitor of class I and II histones deacetylases (HDACs)^4,5^ and is among the family of small-molecule HDACs inhibitors that are known to inhibit cancers.^6–8^ Histone deacetylases regulate gene transcription by deacetylating proteins including histone proteins and transcription factors and resulting in modified chromatin structure. Thus, butyrate may cause a variety of changes in the nucleus, including histone acetylation, DNA methylation and the modification of non-histone proteins. Through these changes, butyrate may elevate the expression level of apoptosis-, inflammatory- and tumor-associated genes.

Reprogrammed metabolism is a hallmark of cancer.^9^ In contrast to normally differentiated cells, which rely primarily on mitochondrial oxidative phosphorylation to generate the energy needed for cellular processes, most cancer cells instead rely on aerobic glycolysis. This type of reprogramming of glycolytic activity versus tricarboxylic acid (TCA) cycle activity is known as the Warburg effect. We identified sirtuin-mediated metabolic acetylation as a conserved regulatory mechanism that coordinates the activities of metabolic pathways.^10,11^ This introduces the possibility that relative glycolytic activity versus TCA cycle activity can be modulated by altering the activity of sirtuins. Remarkably, butyrate was projected and confirmed^12^ to inhibit sirtuins. This introduces the possibility that butyrate, in addition to altering the transcription of genes, may also inhibit tumor growth by reversing the Warburg effect,^13^ a process that is known to induce apoptosis.^14^

The pyruvate dehydrogenase complex (PDC), an enzyme complex that links glycolysis to the TCA cycle, is composed of three enzymatic components: pyruvate dehydrogenase (E1), dihydrolipoamide acetyltransferase (E2) and lipoamide dehydrogenase (E3). The E1 enzyme is a heterotetramer with two alpha and two beta subunits, and the E1 alpha 1 (PDHA1) subunit has a key role in the function of the PDC. PDC is often inactivated in tumor cells and is a possible cause of the Warburg effect.^15^ Conversely, PDC activation could be a possible strategy to prevent or treat cancer.^16^ PDC activity is reportedly regulated by SIRT3-mediated acetylation.^17^ Furthermore, SIRT3 is also involved in the regulation of oxidative phosphorylation through the regulation of complex I of the electron transfer chain. It has been reported that *Sirt3* deletion in mice results in selective inactivation of complex I by altering the function of NDUFA9, a component of complex I.^18^ These studies support a scenario in which butyrate reverses the Warburg effect and induces metabolic stress to induce apoptosis, resulting in increased glycolytic influx and less utilization of TCA cycle intermediates.

## Materials and methods

### Cell lines and cell culture

HEK293T, HeLa and HepG2 cells were maintained in Dulbecco’s modified Eagle’s medium (DMEM) containing glucose (1 g l^−1^), glutamine (2 mm), 10% new-born calf serum, with the exception of HepG2 cells, which were supplemented with 10% fetal bovine serum (FBS). HCT116*p53*^*+/+*^ and HCT116*p53^−/−^* cells were cultured in McCoy’s 5A medium supplemented with 10% FBS and 3 mm glutamine.

### Reagents and antibodies

Butyrate (SIGMA, St Louis, CA, USA), sodium dichloroacetate (SIGMA), nicotinamide (SIGMA), rotenone (MERYER, Shanghai, China), TSA (CST, Danver, MA, USA), PDHA1α-phosphor-Ser293 Antibody (Novus Biologicals, Littleton, CO, USA), PDHA1α antibody (Abmart, Berkeley Heights, NJ, USA), Flag antibody (SIGMA), HA antibody (Santa Cruz, Santa Cruz, CA, USA) and the HIF-1α antibody (ABclone, Shanghai, China) were purchased. The pan-acetylation antibody was generated in-house.^19^

### Isolation of mouse hepatocytes

Primary hepatocytes were isolated from fed adult mice by a modified collagenase method.^20^ The cells were plated at a density of 2.5×10^5^ cells per well on 6-well plates or 1×10^5^ cells per 24-well plates in M199 medium supplemented with 100 U ml^−1^ penicillin, 100 μg ml^−1^ streptomycin, 10% FBS, 500 nm dexamethasone (dex; Sangon Biotech, Shanghai, China), 10 nm insulin (Actrapid, Novo Nordisk, Bagsvaerd, Denmark).

### Immunoprecipitation and western blotting

Cells were lysed on ice in NP-40 buffer (0.1–0.5% NP-40, 150 mm NaCl, 50 mm Tris–HCl (pH 7.5)) supplemented with protease inhibitors (PMSF, Aprotinin, Leupeptin, Pepstatin, Sodium trioxovanadate and sodium fluoride). Flag-tagged proteins were immunoprecipitated and washed in NP-40 buffer three times. Standard western blot procedures were followed. Detection of acetylation by western blotting was achieved by using 50 mm Tris (pH 7.5) with 10% (v/v) Tween 20 and 1% peptone (AMRESCO, Fountain Parkway Solon, OH, USA) as a blocking buffer and we diluted primary and secondary antibodies in 50 mm Tris (pH 7.5) with 0.1% peptone.^10^

### Cell survival and apoptosis assay

Apoptosis of cells was determined by using the annexin V-FITC apoptosis detection kit (eBioscience, San Diego, CA, USA) according to the manufacturer’s instructions on FACScan (BD Pharmigen, Franklin Lakes, NJ, USA). dichloroacetic acid (DCA, 20 mm), rotenone (300 nm) and butyrate (5 mm) treatments were enforced 24 h before cells were subject to analysis.

### PDHA1 activity assay

PDHA1-Flag and PDHB-myc were co-expressed in either HeLa or HEK293T cells. Cells were lysed on ice with 0.5% NP-40 buffer supplemented with protease inhibitors. PDHA1 was immuno-purified using Flag-beads (SIGMA) and washed with 0.1% NP-40 buffer three times. PDHB co-purified with PDHA1 was quantified by myc-western and was used to normalize PDHA1 activity. The PDHA1 activity assay was performed as described previously.^21^ Briefly, in a reaction buffer containing 50 mm KH_2_PO_4_ (pH 7), 1 mm MgCl_2_, 2 mm sodium pyruvate, 0.2 mm thiamine diphosphate and 0.1 mm 2,6-dichlorophenolindophenol (2,6-DCPIP), purified PDHA1/PDHB complex was added to initiate the reaction. The reactions were maintained at 30 °C. The reaction progression was monitored by measuring the reduction of 2,6-DCPIP at 600 nm on a Thermo Fisher BIOMATE 3S (Waltham, MA, USA) spectrophotometer.

### PDC activity assay

The assays were performed using the PDH enzyme activity microplate assay kit (Abcam, Cambridge, UK) according to the manufacturer’s instructions. The total PDC activity of HeLa cells treated with 0.5 mm butyrate for 2 h was detected.

### Liquid chromatography–Mass Spectrometry (LC-MS) analysis

Cells were treated with butyrate (5 mm) for 6 h and DCA (20 mm) for 24 h. Cells were lysed by immediate addition of 1 ml 60% (v/v) pre-chilled (−80 °C) methanol into culture plates, followed by centrifugation for 15 min at 12 000 r.p.m. and 4 °C. The supernatant was collected and dried with a concentrator. After drying, the sample was dissolved in 400 μl of H_2_O. Last, the sample was filtered through a 0.22-μm filter before liquid chromatography–mass spectrometry (LC-MS) analysis.

### Gas chromatography–Mass spectrometry (GC-MS)

Cells were cultured and harvested as in the gas chromatography–mass spectrometry (GC-MS) assay. After centrifugation, the supernatant were lyophilized, followed by oximatation with 20 mg ml^−1^ methoxyamine hydrochloride in pyridine at 30 °C for 60 min. The samples were then derivatized in 80 μl of pyridine and 20 μl of *N*-methyl-*N*-(tert-butyldimethylsilyl) trifluoroacetamide (MTBSTFA) at 70 °C for 30 min. Derivatized samples were filtrated to remove insoluble particles, and 3 μl of each sample was subjected to GC–MS analysis by employing an HP-5MS column (30 m×0.25 mm×0.25 μm) for separation on an Agilent 6890-5973 GC–MS system (Santa Clara, CA, USA).

### Mitochondrion complex I activity assay

Mouse primary hepatocytes and HeLa cells treated with 5 mm butyrate were subjected to this assay. Hepatocytes and HeLa cells were treated for 24 h. The assays were performed using the complex I enzyme activity microplate assay kit (Abcam ab109721) according to the manufacturer’s instructions.

### Glucose uptake assay

This assay was performed using the glucose uptake assay kit (Colorimetric, Abcam ab136955) according to the manufacturer’s instructions. HeLa cells were treated with DCA 20 mm for 24 h and butyrate 5 mm for 24 h.

### RT–PCR

RNA from cultured cells was prepared with TransZol (Trans Gen Biotech, Beijing, China), and cDNA was synthesized from 5 μg of RNA using TransScript First-Strand cDNA synthesis Super Mix (Trans Gen Biotech). The primers used were as follows:[Table tbl1]

### Statistics

Statistically significant differences between samples were determined using an unpaired two-tailed Student’s *t*-test.

## Results

### Butyrate relieves the Warburg effect in cancer cells

**Figure 1:**
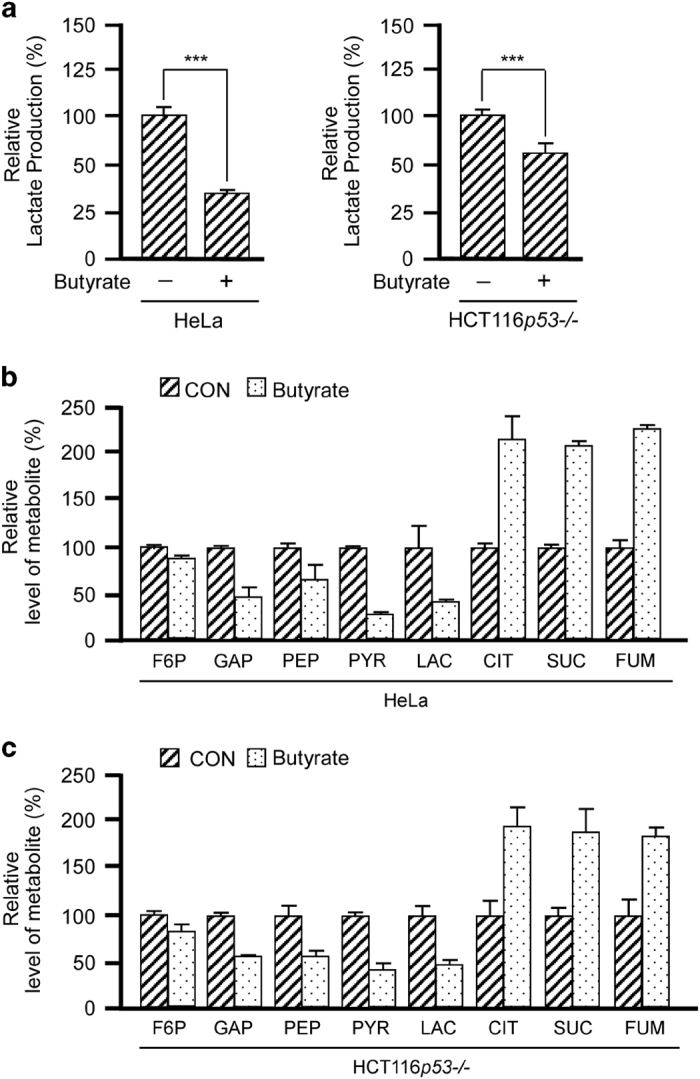
Butyrate relieves the Warburg effect in cancer cells. (**a**) Butyrate treatment decreases lactate production. The lactate levels in the culture media of HeLa and HCT116*p53*^*−/−*^ cells were analyzed with or without butyrate treatments. Shown is the average value of three independent treatments. The lactate levels of untreated HeLa or HCT116*p53*^*−/−*^ cells were arbitrarily set as 100%. (**b**,** c**) Butyrate treatment results in a decrease of glycolytic metabolites and accumulation of citrate. Glycolytic metabolite and TCA intermediary metabolite levels in HeLa cells (**b**) and HCT116*p53*^*−/−*^ cells (**c**) treated with butyrate. Concentrations in untreated cells were set as 100% arbitrarily. Metabolites indistinguishable by LC are shown as the total concentration. The average value of triplicate treatments is shown.

Cancer cells metabolically exert the Warburg effect, which is characterized by avid glucose consumption and lactate production under aerobic conditions rather than the channeling of glucose intermediates into the TCA cycle. We observed that when HeLa and HCT116*p53*^*−/−*^ cells were treated with 5 mm butyrate, which is within the physiological concentration range of colorectal tissue,^6,7^ the lactate production decreased by 65 and 40% compared with that of control groups (Figure 1a). Butyrate treatment in HeLa cells cultured with DMEM media supplemented with a [1,2-^13^C] glucose tracer resulted in decreased concentrations of glycolytic intermediary metabolites, such as fructose 6-phosphate (F6P), glyceraldehyde 3-phosphate (GAP), phosphoenolpyruvate (PEP), pyruvate and lactate (Figure 1b). Surprisingly, the concentrations of intermediary TCA metabolites increased significantly, including citrate, succinate, fumarate and malate (Figure 1b). In the butyrate-treated HCT116*p53*^*−/−*^ cells, we also observed decreased glycolytic metabolites and increased TCA metabolites (Figure 1c). These results indicate that butyrate elevates the influx from glucose into the TCA cycle and mitigates the Warburg effect in cancer cells.

### Butyrate activates PDC

**Figure 2:**
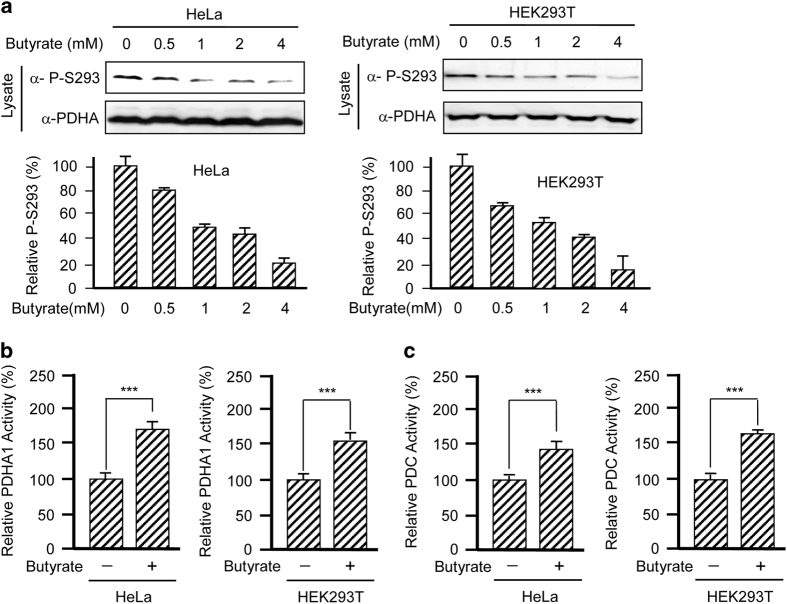
Butyrate activates PDC. (**a**) Butyrate decreased PDHA1 P-S293 levels in a dose-dependent manner. Endogenous PDHA1 P-S293 was probed in lysates of either HeLa or HEK293T cells cultured with different concentrations of butyrate. Reprehensive western blots (left panels) and a summary of densitometric analysis (right graphs) are shown. (**b**) PDHA1 is activated by butyrate. Specific activities of PDHA1 isolated from either HeLa or HEK293T cells cultured with or without butyrate were determined. (**c**) PDC is activated by butyrate. Specific activities of PDC isolated from either HeLa or HEK293T cells cultured with or without butyrate were determined.

Tumor cells often partially inactivate the PDC, which is the enzyme complex that links glycolysis to the TCA cycle, thus partially uncoupling glycolysis from the TCA cycle.^15^ To investigate how butyrate enhances the connection between glycolysis and the TCA cycle in tumor cells, we first tested whether butyrate activates PDC. We treated both HeLa and HEK293T cells with butyrate and analyzed the phosphorylation level of endogenous PDHA1 at serine 293 (P-S293), which is a key indicator of PDHA1 and PDC activity.^22^ We found that butyrate treatment decreased the PDHA1 P-S293 levels in a dose-dependent manner in both cell lines (Figure 2a), suggesting that butyrate may activate PDC. This notion is further supported by measuring PDHA1 activity, the catalytic subunit of PDC and the activity of intact PDC. PDHA1 purified from either HeLa or HEK293T cells cultured with 5 mm butyrate displayed ~50% higher specific catalytic activity compared with PDHA1 obtained from control cells (Figure 2b), which is consistent with the observation that butyrate diminishes P-S293 inhibition of PDHA1. Moreover, the specific activity of PDC isolated from cultured cells was similar to PDHA1 (Figure 2c), suggesting that butyrate activates PDC activity and confirming that butyrate activates PDHA1. These results, together with the observation that butyrate treatment decreases glycolytic metabolic flux and increases TCA metabolic flux (Figures 1b and c), confirm that butyrate activates PDC.

### Butyrate activates PDC by increasing PDHA1 acetylation

**Figure 3:**
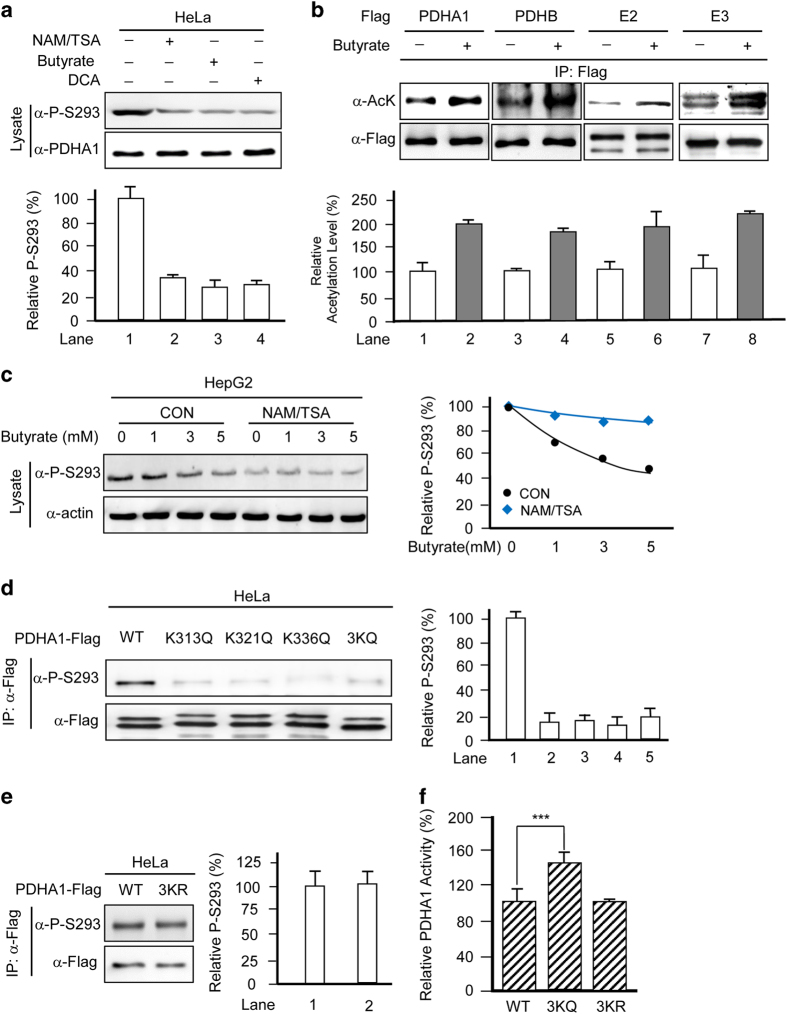
Butyrate activates PDC by increasing PDHA1 acetylation. (**a**) Butyrate decreases PDHA1 P-S293 levels. Endogenous PDHA1 P-S293 levels were probed in lysates of HeLa cells cultured with or without 5 mm butyrate, 20 mm DCA,or NAM (5 mm)/TSA (5 μm). Reprehensive western blots (upper panels) and a summary of densitometric analysis (lower graphs) are shown. (**b**) Acetylation of PDC subunits is enhanced by butyrate treatment. Acetylation levels of overexpressed and immune-purified subunits of PDC from HeLa cells cultured in the absence or presence of 5 mm butyrate were each probed by a pan-acetyllysine antibody. Reprehensive western blots (upper panels) and a summary of densitometric analysis (lower graphs) are shown. (**c**) Deacetylase inhibitors blunt the ability of butyrate to decrease P-S293 levels. Endogenous P-S293 levels of HepG2 cells cultured with or without NAM+TSA were probed by western blotting (left). Relative P-S293 levels were quantified by normalizing the densities of P-S293 to the densities of actin (right). (**d**) Mutations at K131, K321 and K336 residues of PDHA1 decrease PDHA1 P-S293 levels. Levels of P-S293 were determined for purified wild-type, K131Q, K321Q, K336Q and 3KQ PDHA1 mutants, respectively. Reprehensive western blots (left panels) and a summary of densitometric analysis (right graphs) are shown. (**e**) The P-S293 levels of 3KR are comparable to that of wild-type PDHA1. P-S293 levels of wild-type and 3KR mutant PDHA1 were determined. Reprehensive western blots (left panels) and a summary of densitometric analysis (right graphs) are shown. (**f**) Acetylation mimetic mutation activates PDHA1. Specific activities of wild-type, 3KQ and 3KR PDHA1 mutants were analyzed. The specific activity of wild type was set as 100%, and the relative activities of mutants were compared with those of the wild-type enzyme.

Previous reports have shown that acetylation inactivates PDC.^17^ We thus measured the changes in acetylation and activity of PDHA1 and PDC on butyrate treatment. Like DCA, a well-established PDC activator, treatment with butyrate leads to reduced PDHA1 P-S293 level (Figure 3a). Meanwhile, treating the cultured cells with a mix of general deacetylase inhibitors, nicotinamide/trichostatin A (NAM/TSA), also reduced PDHA1 P-S293 levels (Figure 3a). These results suggested that acetylation activates rather than inactivates PDC. This finding was further substantiated by the observation that butyrate treatment increased the acetylation of all PDC subunits, namely, E1 (PDHA and PDHB), E2 (DLAT) and E3 (DLD) (Figure 3b). Moreover, the ability of butyrate to decrease P-S293 levels was abrogated in the presence of NAM/TSA (Figure 3c), further confirming that acetylation activates PDC. To elucidate the molecular mechanisms of butyrate-mediated PDHA1 acetylation and activation, we screened all known PDHA1 acetylation sites to assess their ability to decrease P-S293 levels. Among a number of acetylation sites we identified in PDHA1, we also substituted lysine 313 (K313), lysine 321 (K321) and lysine 336 (K336) with the acetylation mimetic glutamine (K/Q) and found that the PDHA1 P-S293 levels were reduced. On converting all three lysine residues to glutamine (PDHA1^3KQ^), we observed a synergistic decrease in PDHA1 P-S293 levels (Figure 3d). In contrast, replacing these lysines with non-acetylable lysine memetic arginines (PDHA1^3KR^) resulted in negligible changes in P-S293 levels (Figure 3e). Furthermore, purified PDHA1^3KQ^ was 42% more active than wild-type PDHA1, whereas the activity of PDHA1^3KR^ was comparable to that of wild-type PDHA1 (Figure 3f). These results confirmed that these three lysine residues are responsible for acetylation-mediated activation of PDHA1.

### Butyrate increases PDHA1 acetylation by inhibiting SIRT3

**Figure 4:**
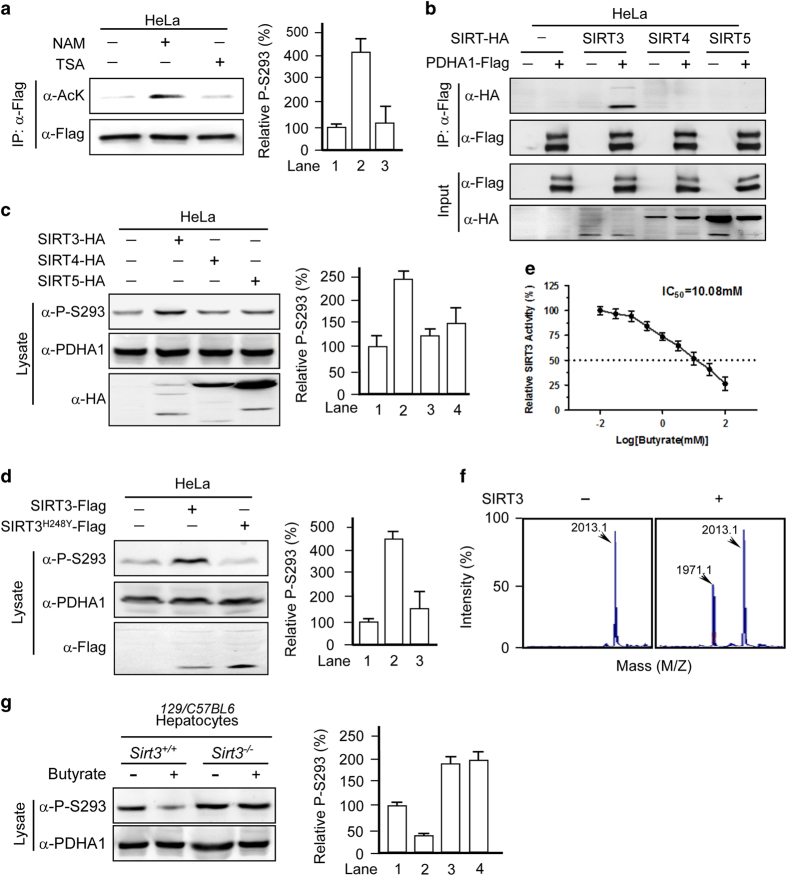
Butyrate increases PDHA1 acetylation by inhibiting SIRT3. (**a**) NAM treatment increases acetylation of PDHA1. Acetylation levels of PDHA1-Flag expressed and isolated from HeLa cells treated with either NAM or TSA were determined. Reprehensive western blots (left panels) and a summary of densitometric analysis (right graphs) are shown. (**b**) SIRT3 interacts with PDHA1. Flag-tagged PDHA1 was co-expressed with HA-tagged SIRT3, SIRT4 and SIRT5, respectively, in HeLa cells. Interactions were determined by co-immunoprecipitation. (**c**) SIRT3 increases PDHA1 P-S293 levels. Endogenous PDHA1 P-S293 levels were determined in lysates of HeLa cells overexpressing SIRT3, SIRT4 or SIRT5. Reprehensive western blots (left panels) and a summary of densitometric analysis (right graphs) are shown. (**d**) SIRT3 deacetylase activity is required for P-S293 regulation. Endogenous levels of P-S293 were determined when either SIRT3 or catalytic-dead SIRT3^H248Y^ was overexpressed in HeLa cells. Reprehensive western blots (left panels) and a summary of densitometric analysis (right graphs) are shown. (**e**,** f**) Butyrate inhibits SIRT3 activity *in vitro*. A synthetically acetylated K336-containing PDHA1 peptide was subjected to *in vitro* deacetylation by recombinant SIRT3, and the resulting products were analyzed by MS. Numbers denote the mass of detected peptides. Deacetylated peptides (*M*/*Z*: 1971.1) were detected in the SIRT3 supplemented reaction system. (**g**) Butyrate treatment did not change PDHA1 P-S293 levels in *Sirt3*^−/−^ mouse hepatocytes. Endogenous PDHA1 P-S293 levels were measured in hepatocytes from *Sirt3*^−/−^ mice and control mice treated with or without butyrate. Reprehensive western blots (left panels) and a summary of densitometric analysis (right graphs) are shown.

To verify that acetylation activates PDHA1, we identified the corresponding deacetylase for PDHA1. PDHA1 acetylation could only be increased by treatment with the class III deacetylase inhibitor NAM but not by treatment with the class I, II and VI deacetylase inhibitor TSA (Figure 4a), suggesting that PDHA1 is deacetylated by an NAD^+^-dependent sirtuin family deacetylase. Given that PDHA1 is located in the mitochondria, we tested the ability of mitochondrial sirtuins (SIRT3, SIRT4 and SIRT5) to alter endogenous P-S293. In the co-immunoprecipitation assays, we found that only SIRT3 binds to PDHA1 (Figure 4b). Moreover, overexpression of SIRT3, but not that of SIRT4 or SIRT5, increased P-S293 levels (Figure 4c), suggesting that SIRT3 is a deacetylase for PDHA1. Furthermore, overexpression of SIRT3, but not a catalytically dead mutant SIRT3^H248Y^,^23^ increased P-S293 levels (Figure 4d), demonstrating that increased SIRT3 catalytic activity is the cause of the increased P-S293 levels in this system. In addition, butyrate inhibits SIRT3 activity, with a median inhibitory concentration (IC_50_) of 10.08 mm *in vitro* (Figure 4e). Next, we confirmed the ability of SIRT3 to deacetylate PDHA1 by demonstrating that recombinant SIRT3 deacetylates a synthetic K336^Ac^-containing PDHA1 peptide *in vitro* (Figure 4f). Importantly, butyrate treatment failed to induce PDHA1 acetylation and decreased P-S293 levels in hepatocytes from *Sirt3*^*−/−*^ mice (Figure 4g). These data demonstrate that SIRT3 is the major, if not only, deacetylase for PDHA1.

### Butyrate inhibits complex I by inhibiting SIRT3

**Figure 5:**
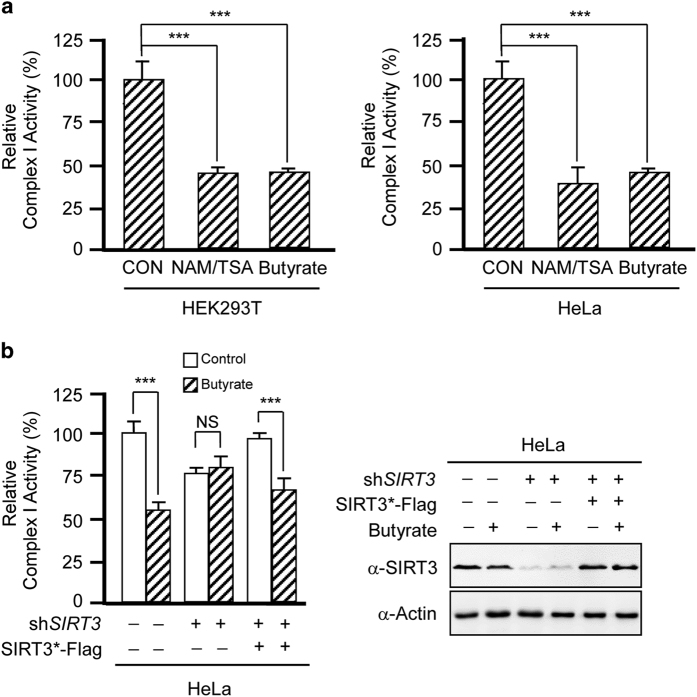
Butyrate inhibits complex I by inhibiting SIRT3. (**a**) Butyrate and NAM/TSA treatment inactivates complex I. Complex I activity was measured in lysates of either HeLa or HEK293T cells with and without butyrate or NAM/TSA treatment. (**b**) Butyrate-mediated inhibition of complex I activity is dependent on SIRT3. Complex I activity was measured in lysates of HeLa cells after *SIRT3* knockdown and silent mutated SIRT3 (SIRT3*) reintroduction through transfection. The SIRT3 expression levels were shown in the right panel.

SIRT3 has an important role in regulating energy homeostasis. It was previously reported that *Sirt3* knockout in mice selectively inactivates complex I by altering the function of NDUFA9, a component of complex I.^18^ We observed that treatment with both butyrate and deacetylase inhibitors decreased the activity of complex I in either HeLa or HEK293T cells (Figure 5a), suggesting that the activity of human complex I is negatively regulated by acetylation. Furthermore, knockdown of *SIRT3* in HeLa cells reduced complex I activity and blunted the ability of butyrate to inhibit the activity of complex I, whereas SIRT3 reintroduction increased complex I activity and partially restored the ability of butyrate to inhibit complex I (Figure 5b). Altogether, these results confirm that butyrate inactivates complex I by inhibiting SIRT3.

### Butyrate promotes glycolytic cell apoptosis by activating PDC and inhibiting complex I

**Figure 6:**
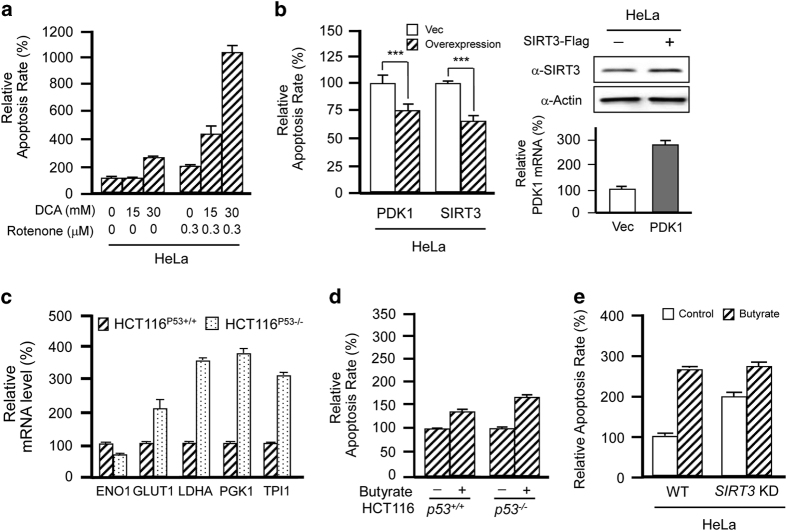
Butyrate promotes glycolytic cell apoptosis by activating PDC and inhibiting complex I. (**a**) Rotenone potentiates DCA-induced apoptosis. Apoptosis rates of HeLa cells were analyzed following treatment with DCA, rotenone or both, as indicated. (**b**) Overexpression of PDK1 and SIRT3 decreases apoptosis. Apoptosis rates of HeLa cells and HeLa cells overexpressing PDK1 or SIRT3 were analyzed. The transfection efficiencies were shown in the right panels. (**c**) HCT116*p53−/−* cells are more glycolytic than HCT116*p53+/+* cells. Relative mRNA levels of glycolytic enzymes were analyzed for HCT116*p53−/−* cells and HCT116*p53*^*+/+*^ cells. (**d**) Butyrate treatment promotes higher levels of apoptosis in HCT116*p53*^*−/−*^ cells compared with HCT116*p53*^*+/+*^ cells. Apoptosis rates of HCT116*p53*^*−/−*^ and HCT116*p53*^*+/+*^ cells were determined with and without butyrate treatments. Untreated values were set as 100% arbitrarily. (**e**) *SIRT3* knockdown reduced butyrate-induced apoptosis. Apoptosis rates of HeLa cells and HeLa cells after *SIRT3* knocked down were analyzed in the presence or absence of butyrate.

Complex I inhibition by compounds such as by rotenone induces apoptosis.^24^ Butyrate treatment also increases cell apoptosis.^25–28^ To simulate the apoptosis-inducing effects of butyrate-induced PDC activation and complex I inhibition, we measured the apoptosis rate in HeLa cells when complex I was inhibited by rotenone and PDC was activated by DCA. Either DCA treatment or rotenone treatment lead to mild apoptosis. Surprisingly, rotenone-treated cells were extremely sensitive to the activation of PDC by DCA, and we observed increased rates of apoptosis (Figure 6a). In the presence of 300 nm rotenone, 15 mm DCA treatment increased apoptosis by more than 100% and 30 mm DCA treatment increased apoptosis by more than 400% (Figure 6a), suggesting that PDC activation-induced apoptosis is potentiated by complex I inhibition. Moreover, treatment with 300 nm rotenone and inactivation of PDC by either overexpression of PDK1 or SIRT3 decreased apoptosis in HeLa cells (Figure 6b). Taken together, these results demonstrate that when complex I is inhibited, increased influx of TCA intermediates causes severe apoptosis. Therefore, we reasoned that glycolytic tumor cells would be more prone to butyrate-induced apoptosis than normal cells because more TCA cycle metabolites would be influx after complex I inhibition. Because HCT116*p53*^*−/−*^ cells are more glycolytic than HCT116*p53*^*+/+*^ cells due to the lack of negative glycolytic regulation by p53,^29^ we next compared the apoptotic rates of HCT116*p53*^*+/+*^ and HCT116*p53*^*−/−*^ cells in response to butyrate treatment (Figure 6c). At a concentration of 10 mm, butyrate treatment induced nearly 180% more apoptosis in HCT116*p53*^*−/−*^ cells compared with only 47% more apoptosis in HCT116*p53*^*+/+*^ cells (Figure 6d), confirming that butyrate preferentially induces apoptosis in glycolytic cells and explaining why butyrate inhibits the growth of HCT116*p53*^*−/−*^ cells more effectively than HCT116*p53*^*+/+*^ cells.^30^ Finally, SIRT3 knockdown increased apoptosis in HeLa cells but blunted butyrate-induced apoptosis from ~160 to ~50% (Figure 6e), confirming that butyrate induces apoptosis via the inhibition of SIRT3.

## Discussion

**Figure 7:**
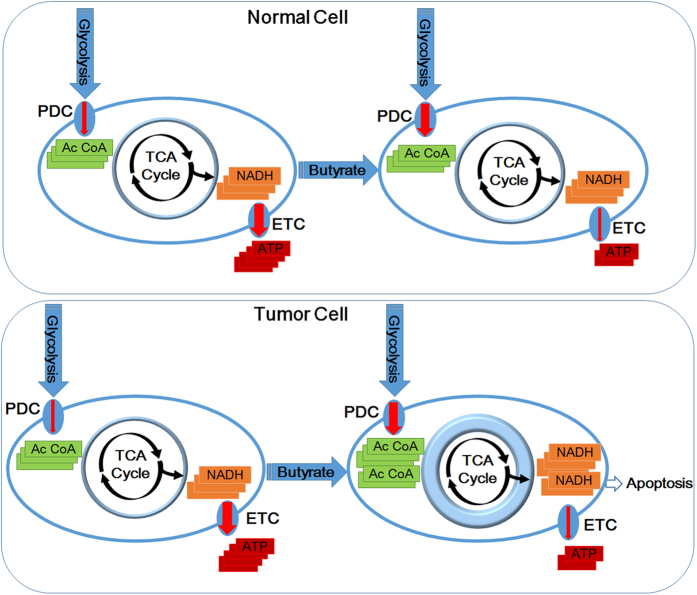
Schematic diagram of the tumor-preventive mechanisms of butyrate. Butyrate causes concomitant activation of PDC and inactivation of complex I, and the inconsistency between increased influx of TCA intermediates and decreased ability to convert TCA intermediates to ATP causes higher apoptosis in highly glycolytic tumor cells.

Our study is the first to report that butyrate exerts anticancer efficacy by inhibiting the type III deacetylase SIRT3. *In vitro*, butyrate treatment inhibited the ability of SIRT3 to deacetylate a synthetic acetylated K336-containing PDHA1 peptide. In cultured cells, butyrate treatment increased the pan-acetylation levels of mitochondrial proteins PDHA1 and complex I. At molecular level, butyrate treatment induced acetylation on the K313, K321 and K336 residues of PDHA1, decreased PDHA1 P293 levels, activated PDC and increased the influx of glucose intermediates into mitochondria. Meanwhile, butyrate treatment induced acetylation on complex I, thus inactivating complex I and preventing TCA intermediates from being converted to ATP. The net outcome of butyrate treatment is therefore to create metabolic stress that promotes apoptosis and inhibits the growth of cancer cells (Figure 7).

Butyrate is a primary end-product of microbial fermentation of dietary fiber in the intestine. Our findings provide plausible explanations for the importance of balancing the intestinal microbiome and consuming dietary fibers. We have also shown that butyrate could reduce the number of transformed cells by promoting their apoptosis through concerted activation of PDC and inactivation of complex I. Our findings at least partially explain the anti-tumor effects of butyrate and can be further explored. For example, exaggerating complex I inhibition and PDC activation to induce more severe apoptosis in highly glycolytic cancer cells could serve as a cancer preventive or treatment strategy.

## Figures and Tables

**Table 1 tbl1:** 

*Genes*	*Forward (5′–3′)*	*Reverse (5′–3′)*
*ACTB*	TCCCTGGAGAAGAGCTACG	GTAGTTTCGTGGATGCCACA
*GLUT1*	TGGCTACAACACTGGAGTCATCA	GGACCCATGTCTGGTTGTAGAACT
*GPI*	AAACATGTTCGAATTATGGGA	GCTCGAAGTTGTCAAAACCC
*HK2*	CTTCTTCACGGAGCTCAACC	AAGCCCTTTCTCCATCTCCT
*ENO1*	TACGTTCACCTCGGTGTCTG	CCTGGCATGGATCTTGAGAA
*TPI1*	AGCTCATCGGCACTCTGAAC	CCACAGCAATCTTGGGATCT
*PGK1*	ATGGATGAGGTGGTGAAAGC	CAGTGCTCACATGGCTGACT
*LDHA*	GGCCTGTGCCATCAGTATCT	GGAGATCCATCATCTCTCCC
*P21*	TGTCCGTCAGAACCCATGC	AAAGTCGAAGTTCCATCGCTC

## References

[bib1] Shin H, Lee YS, Lee YC. Sodium butyrate-induced DAPK-mediated apoptosis in human gastric cancer cells. Oncol Rep 2012; 27: 1111–1115. 22160140 10.3892/or.2011.1585 PMC3583600

[bib2] Fung KY, Brierley GV, Henderson S, Hoffmann P, McColl SR, Lockett T et al. Butyrate-induced apoptosis in HCT116 colorectal cancer cells includes induction of a cell stress response. J Proteome Res 2011; 10: 1860–1869. 21235278 10.1021/pr1011125

[bib3] Barrasa JI, Santiago-Gomez A, Olmo N, Lizarbe MA, Turnay J. Resistance to butyrate impairs bile acid-induced apoptosis in human colon adenocarcinoma cells via up-regulation of Bcl-2 and inactivation of Bax. Biochim Biophys Acta 2012; 1823: 2201–2209. 22917577 10.1016/j.bbamcr.2012.08.008

[bib4] Sekhavat A, Sun JM, Davie JR. Competitive inhibition of histone deacetylase activity by trichostatin A and butyrate. Biochem Cell Biol 2007; 85: 751–758. 18059533 10.1139/o07-145

[bib5] Davie JR. Inhibition of histone deacetylase activity by butyrate. J Nutr 2003; 133: 2485S–2493S. 12840228 10.1093/jn/133.7.2485S

[bib6] Aune D, Chan DS, Lau R, Vieira R, Greenwood DC, Kampman E et al. Dietary fibre, whole grains, and risk of colorectal cancer: systematic review and dose-response meta-analysis of prospective studies. BMJ 2011; 343: d6617. 22074852 10.1136/bmj.d6617 PMC3213242

[bib7] Buda A, Qualtrough D, Jepson MA, Martines D, Paraskeva C, Pignatelli M. Butyrate downregulates alpha2beta1 integrin: a possible role in the induction of apoptosis in colorectal cancer cell lines. Gut 2003; 52: 729–734. 12692060 10.1136/gut.52.5.729 PMC1773640

[bib8] Ganapathy V, Thangaraju M, Prasad PD, Martin PM, Singh N. Transporters and receptors for short-chain fatty acids as the molecular link between colonic bacteria and the host. Curr Opin Pharmacol 2013; 13: 869–874. 23978504 10.1016/j.coph.2013.08.006

[bib9] Hanahan D, Weinberg RA. Hallmarks of cancer: the next generation. Cell 2011; 144: 646–674. 21376230 10.1016/j.cell.2011.02.013

[bib10] Zhao S, Xu W, Jiang W, Yu W, Lin Y, Zhang T et al. Regulation of cellular metabolism by protein lysine acetylation. Science 2010; 327: 1000–1004. 20167786 10.1126/science.1179689 PMC3232675

[bib11] Wang Q, Zhang Y, Yang C, Xiong H, Lin Y, Yao J et al. Acetylation of metabolic enzymes coordinates carbon source utilization and metabolic flux. Science 2010; 327: 1004–1007. 20167787 10.1126/science.1179687 PMC4183141

[bib12] Yoo DY, Kim DW, Kim MJ, Choi JH, Jung HY, Nam SM et al. Sodium butyrate, a histone deacetylase Inhibitor, ameliorates SIRT2-induced memory impairment, reduction of cell proliferation, and neuroblast differentiation in the dentate gyrus. Neurol Res 2015; 37: 69–76. 24963697 10.1179/1743132814Y.0000000416

[bib13] Poteet E, Choudhury GR, Winters A, Li W, Ryou MG, Liu R et al. Reversing the Warburg effect as a treatment for glioblastoma. J Biol Chem 2013; 288: 9153–9164. 23408428 10.1074/jbc.M112.440354 PMC3610988

[bib14] Arora R, Schmitt D, Karanam B, Tan M, Yates C, Dean-Colomb W. Inhibition of the Warburg effect with a natural compound reveals a novel measurement for determining the metastatic potential of breast cancers. Oncotarget 2015; 6: 662–678. 25575825 10.18632/oncotarget.2689 PMC4359247

[bib15] McFate T, Mohyeldin A, Lu H, Thakar J, Henriques J, Halim ND et al. Pyruvate dehydrogenase complex activity controls metabolic and malignant phenotype in cancer cells. J Biol Chem 2008; 283: 22700–22708. 18541534 10.1074/jbc.M801765200 PMC2504897

[bib16] Papandreou I, Goliasova T, Denko NC. Anticancer drugs that target metabolism: Is dichloroacetate the new paradigm? Int J Cancer 2011; 128: 1001–1008. 20957634 10.1002/ijc.25728

[bib17] Fan J, Shan C, Kang HB, Elf S, Xie J, Tucker M et al. Tyr phosphorylation of PDP1 toggles recruitment between ACAT1 and SIRT3 to regulate the pyruvate dehydrogenase complex. Mol Cell 2014; 53: 534–548. 24486017 10.1016/j.molcel.2013.12.026 PMC3943932

[bib18] Ahn BH, Kim HS, Song S, Lee IH, Liu J, Vassilopoulos A et al. A role for the mitochondrial deacetylase Sirt3 in regulating energy homeostasis. Proc Natl Acad Sci USA 2008; 105: 14447–14452. 18794531 10.1073/pnas.0803790105 PMC2567183

[bib19] Guan KL, Yu W, Lin Y, Xiong Y, Zhao S. Generation of acetyllysine antibodies and affinity enrichment of acetylated peptides. Nat Protoc 2010; 5: 1583–1595. 21085124 10.1038/nprot.2010.117 PMC4889332

[bib20] Foretz M, Hébrard S, Leclerc J, Zarrinpashneh E, Soty M, Mithieux G et al. Metformin inhibits hepatic gluconeogenesis in mice independently of the LKB1/AMPK pathway via a decrease in hepatic energy state. J Clin Invest 2010; 120: 2355–2369. 20577053 10.1172/JCI40671 PMC2898585

[bib21] Nemeria N, Yan Y, Zhang Z, Brown AM, Arjunan P, Furey W et al. Inhibition of the *Escherichia coli* pyruvate dehydrogenase complex E1 subunit and its tyrosine 177 variants by thiamin 2-thiazolone and thiamin 2-thiothiazolone diphosphates. Evidence for reversible tight-binding inhibition. J Biol Chem 2001; 276: 45969–45978. 11583990 10.1074/jbc.M104116200

[bib22] Rardin MJ, Wiley SE, Naviaux RK, Murphy AN, Dixon JE. Monitoring phosphorylation of the pyruvate dehydrogenase complex. Anal Biochem 2009; 389: 157–164. 19341700 10.1016/j.ab.2009.03.040 PMC2713743

[bib23] Hirschey MD, Shimazu T, Goetzman E, Jing E, Schwer B, Lombard DB et al. SIRT3 regulates mitochondrial fatty-acid oxidation by reversible enzyme deacetylation. Nature 2010; 464: 121–125. 20203611 10.1038/nature08778 PMC2841477

[bib24] Li N, Ragheb K, Lawler G, Sturgis J, Rajwa B, Melendez JA et al. Mitochondrial complex I inhibitor rotenone induces apoptosis through enhancing mitochondrial reactive oxygen species production. J Biol Chem 2003; 278: 8516–8525. 12496265 10.1074/jbc.M210432200

[bib25] Clarke JM, Topping DL, Bird AR, Young GP, Cobiac L. Effects of high-amylose maize starch and butyrylated high-amylose maize starch on azoxymethane-induced intestinal cancer in rats. Carcinogenesis 2008; 29: 2190–2194. 18701436 10.1093/carcin/bgn192 PMC2577140

[bib26] Fung KY, Cosgrove L, Lockett T, Head R, Topping DL. A review of the potential mechanisms for the lowering of colorectal oncogenesis by butyrate. Br J Nutr 2012; 108: 820–831. 22676885 10.1017/S0007114512001948

[bib27] Tailor D, Hahm ER, Kale RK, Singh SV, Singh RP. Sodium butyrate induces DRP1-mediated mitochondrial fusion and apoptosis in human colorectal cancer cells. Mitochondrion 2014; 16: 55–64. 24177748 10.1016/j.mito.2013.10.004 PMC4004730

[bib28] Wang L, Luo HS, Xia H. Sodium butyrate induces human colon carcinoma HT-29 cell apoptosis through a mitochondrial pathway. J Int Med Res 2009; 37: 803–811. 19589263 10.1177/147323000903700323

[bib29] Yeung SJ, Pan J, Lee MH. Roles of p53, MYC and HIF-1 in regulating glycolysis—the seventh hallmark of cancer. Cell Mol Life Sci 2008; 65: 3981–3999. 18766298 10.1007/s00018-008-8224-x PMC11131737

[bib30] Donohoe DR, Collins LB, Wali A, Bigler R, Sun W, Bultman SJ et al. The Warburg effect dictates the mechanism of butyrate-mediated histone acetylation and cell proliferation. Mol Cell 2012; 48: 612–626. 23063526 10.1016/j.molcel.2012.08.033 PMC3513569

